# Identification of *Pou5f1*, *Sox2*, and *Nanog *downstream target genes with statistical confidence by applying a novel algorithm to time course microarray and genome-wide chromatin immunoprecipitation data

**DOI:** 10.1186/1471-2164-9-269

**Published:** 2008-06-03

**Authors:** Alexei A Sharov, Shinji Masui, Lioudmila V Sharova, Yulan Piao, Kazuhiro Aiba, Ryo Matoba, Li Xin, Hitoshi Niwa, Minoru SH Ko

**Affiliations:** 1 Developmental Genomics and Aging Section, Laboratory of Genetics, National Institute on Aging, NIH, Baltimore, MD 21224, USA; 2 Laboratory of Pluripotent Cell Studies, RIKEN Center for Developmental Biology, Kobe 650-0047, Japan

## Abstract

**Background:**

Target genes of a transcription factor (TF) *Pou5f1 *(*Oct3/4 *or *Oct4*), which is essential for pluripotency maintenance and self-renewal of embryonic stem (ES) cells, have previously been identified based on their response to *Pou5f1 *manipulation and occurrence of Chromatin-immunoprecipitation (ChIP)-binding sites in promoters. However, many responding genes with binding sites may not be direct targets because response may be mediated by other genes and ChIP-binding site may not be functional in terms of transcription regulation.

**Results:**

To reduce the number of false positives, we propose to separate responding genes into groups according to direction, magnitude, and time of response, and to apply the false discovery rate (FDR) criterion to each group individually. Using this novel algorithm with stringent statistical criteria (FDR < 0.2) to a compendium of published and new microarray data (3, 6, 12, and 24 hr after *Pou5f1 *suppression) and published ChIP data, we identified 420 tentative target genes (TTGs) for *Pou5f1*. The majority of TTGs (372) were down-regulated after *Pou5f1 *suppression, indicating that the *Pou5f1 *functions as an activator of gene expression when it binds to promoters. Interestingly, many activated genes are potent suppressors of transcription, which include polycomb genes, zinc finger TFs, chromatin remodeling factors, and suppressors of signaling. Similar analysis showed that *Sox2 *and *Nanog *also function mostly as transcription activators in cooperation with *Pou5f1*.

**Conclusion:**

We have identified the most reliable sets of direct target genes for key pluripotency genes – *Pou5f1*, *Sox2*, and *Nanog*, and found that they predominantly function as activators of downstream gene expression. Thus, most genes related to cell differentiation are suppressed indirectly.

## Background

Identification of direct targets of transcription factors (TFs) is a necessary step to reconstruct gene regulatory networks in living cells. Although traditional single-gene experiments (e.g., assaying promoter activity using a promoter-reporter gene construct) remain most reliable in testing direct targets, there is also a need for high-throughput approaches that would allow one to detect the majority of most important target genes. Two experimental methods contribute most to such a high-throughput search of target genes: gene expression profiling of TF-manipulated cells and genome-wide chromatin immunoprecipitation (ChIP) assay. However, expression profiling may yield many genes that respond indirectly, whereas ChIP may yield many non-functional binding sites, i.e., binding sites that were detected by ChIP, but were not functional for transcriptional regulation. Therefore, the state of the art is to take the intersection between these two sets of genes (i.e. select genes that responded to the manipulation of a TF and have binding sites) and consider it as a set of tentative target genes (TTGs) [1,2]. However, the intersection of these sets of genes may still contain numerous false positives – genes that respond indirectly and have non-functional ChIP-binding sites. Another problem is that there is no method to statistically quantify the proportion of false positives in the set of TTGs.

**Figure 1:**
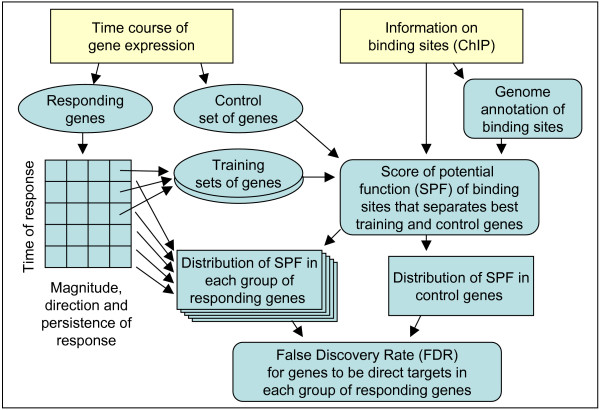
**A flow chart showing algorithm used to identify tentative target genes for a transcription factor.** The algorithm includes the optimization of Scores of Potential Function (SPF) based on the comparison of training and control sets of genes, and the estimation of False Discovery Rates (FDR) within individual groups of responding genes.

To address these issues, we developed a new method to identify TTGs, which reduces the proportion of false positives by applying the False Discovery Rate (FDR) criterion to individual groups of genes that differ in the direction, magnitude, and time of response to the manipulation of a TF. The computational strategy included optimization of Scores of Potential Function (SPF) of binding sites that separated best the training and control sets of genes, and estimation of the FDR from the frequency distribution of SPF among control genes (Fig. 1).

This method is applied here to the mouse *Pou5f1 *(*Oct3/4*, *Oct4*) gene which is the major TF that controls self-renewal and pluripotency in ES cells [3,4]. Lists of potential target genes of POU5F1 were recently generated using chromatin immunoprecipitation (ChIP) and gene expression profiling of cells with suppressed *Pou5f1 *transcription [1,5]. Most studies used shRNA for *Pou5f1 *suppression [1,5], but these methods can generate off-target effects, and gene repression is often weak. In these studies, expression profiling was carried out with 1 day intervals which limited the temporal resolution in detecting gene response. Matoba et al. [2] increased the reliability of prediction of *Pou5f1 *primary targets by using a tet-inducible system to suppress *Pou5f1*. This method eliminated false-positives related to potential off-target effects of shRNA used to suppress *Pou5f1 *in earlier studies. However, gene expression was still measured in 1 day intervals, and microarrays did not include all the mouse genes. In this paper we present a new microarray experiment with the same tet-inducible system but with multiple time points within 24 hr to capture early responses to *Pou5f1 *suppression. These data were analyzed together with published genome-wide ChIP data [1]. We found that most TTGs of *Pou5f1 *in ES cells were activated by *Pou5f1 *and only a limited number of genes were suppressed, which implies that the main function of *Pou5f1 *binding to promoters of target genes is activation of gene expression rather than suppression. The same method was then applied to find target genes of *Sox2 *and *Nanog *based mostly on previously published data. A list of data sets used in this study is shown in Additional file 1. Because the interaction between POU5F1, SOX2, and NANOG is supported by immunoprecipitation, functional analysis, and co-localization of binding sites [6,1-10], we explored the relationships between their target genes. These results are discussed in relation to the mechanism of pluripotency maintenance in ES cells.

## Results and Discussion

### 1. Time course microarray data

We previously reported time course microarray data of mouse ES cells, in which the level of *Pou5f1 *expression was reduced in tetracycline (Tet)-controllable manner [2]. Global gene expression profiles were obtained from ES samples at 24, 48, 72, 96, and 120 hr [2]. To obtain earlier effects of *Pou5f1 *repression, here we used the same ZHBTc4 ES cells [4] and measured the global gene expression profiles at 0, 3, 6, 12, and 24 hr after adding Tet. Because the 24 hr time point was present in both experiments, the microarray data at 24 hr was used to merge two data sets (see Methods) (Additional file 2). In ZHBTc4 ES cells, the level of *Pou5f1 *mRNA was reduced to one-thirds within 3 hrs after adding Tet, whereas the same reduction of POU5F1 protein level was observed in 6 hrs (Additional file 3). The cells show no morphological changes associated with differentiation during the first 24 hrs after adding Tet, but the cells begin to be flattened after 48 hrs [4]. We found 6197 genes with a statistically significant (FDR <= 0.05, fold change >= 1.5) response to *Pou5f1 *suppression (Additional file 4). A subset of these genes with > 2 fold change (*N *= 2600) overlapped reasonably (*N *= 1319, 50.7%) with gene lists identified earlier using *Pou5f1 *knockdown with shRNA [5,1] (Additional file 5A). The majority (*N *= 1185, 96.7%) of 1225 genes common between our study and the published study [1], changed their expression in the same direction after *Pou5f1 *suppression, which was significantly greater than expected from random matches (chi-square = 685, p = 10^-150^) (Additional file 5B). Discrepancies between data sets may be explained by off-target effects of shRNA in earlier studies and by a larger coverage of genes in our microarrays. Genes that were previously considered targets of POU5F1 [1], but had no response to *Pou5f1 *suppression in our experiments (*N *= 487), were not artifacts of microarray design, because the majority of these genes (*N *= 442) had functional oligos in our microarray that showed statistically significant differential expression in earlier experiments [2,11].

**Figure 2:**
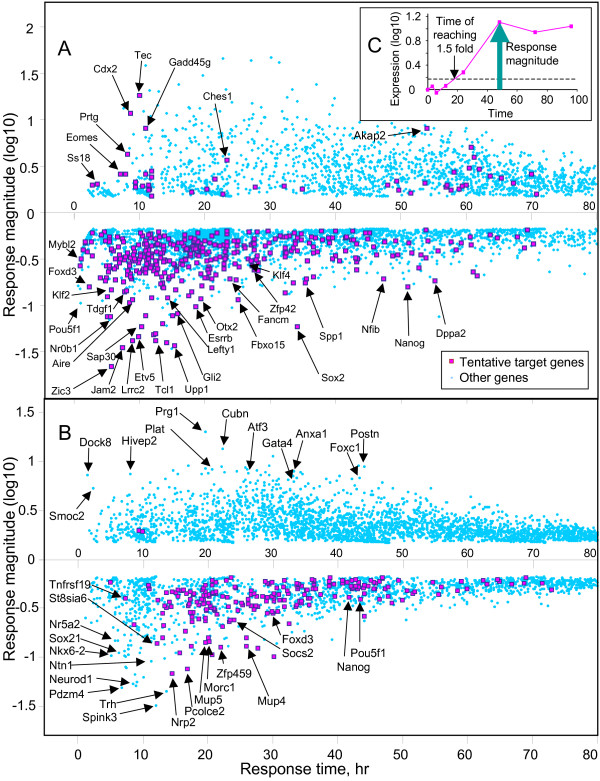
**Gene expression responses to the suppression of *Pou5f1 *(A) or *Sox2 *(B) expression in ES cells.** Time and magnitude of responses were estimated from the time course microarray data using 1.5-fold expression changes as a threshold (C), and then used as coordinates in a scatter-plot. Tentative target genes were identified as shown in Fig 1.

Although several methods to analyze time course microarray data have been reported (e.g. clustering [12,13], splines [14,15]), we decided to characterize the pattern of gene expression in a simpler way by 2 major parameters: the time of response when it exceeded 1.5-fold change threshold, and the magnitude of response measured by the maximum logratio of expression change (Fig. 2C, Additional file 4) (see Materials and methods for details). The scatterplot of magnitude of response versus time of response for all genes with statistically significant change of expression (FDR <= 0.05, fold change >= 1.5) shows the global picture of gene expression change after manipulation of the *Pou5f1 *(Fig. 2A). The initial response to *Pou5f1 *suppression (< 24 hr) is characterized mostly by down-regulation of many genes. It is followed by the wave of gene up-regulation which becomes more intense after 36 hr. Genes with a strongest response had a tendency to be activated or suppressed earlier than genes with a weaker response. Earliest effects were suppression of TFs *Foxd3*, *Mybl2*, *Zic3*, *Klf2*, and *Nr0b1*. Later but still within 12 hr we observed activation of TFs that are expressed in trophectoderm (*Eomes, Cdx2, Gata2, Irx3*). Interestingly, genes that are considered important for pluripotency in ES cells (*Sox2, Nanog, Klf4, Zfp42*) responded with a considerable delay (> 24 hr).

Gene expression profiling of ES cells after suppression of *Sox2 *was carried out using the same protocol (Tet-inducible transgene cell line 2TS22C; the same array platform) as the experiment on *Pou5f1 *[16]. Thus, results on *Sox2 *(Fig. 2B, Additional file 6) were fully compatible with that for *Pou5f1 *experiment.

**Figure 3:**
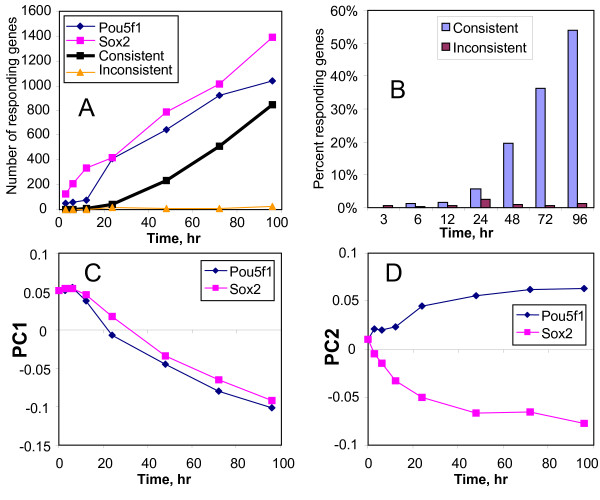
**Comparison of gene expression responses to suppression of *Pou5f1 *and *Sox2*:** (A) number of genes with > 2 fold change in gene expression after suppression of *Pou5f1 *and *Sox2*; (B) proportion of genes with consistent responses (i.e., common genes whose expression changed in the same direction) and inconsistent responses (i.e., common genes whose expression changed in the opposite direction) to *Pou5f1 *and *Sox2 *suppression among the combined list of genes that responded by > 2 fold to either of these transcription factors; (C-D) Principal Component Analysis (PCA) of gene expression response to the suppression of *Pou5f1 *and *Sox2 *based on combined data.

The proportion of genes that responded to the suppression of *Pou5f1 *and *Sox2 *increased in a similar manner over time (Fig 3A), and the proportion of common genes also increased with time (Fig. 3A, B). Principal Component Analysis (PCA) showed that gene expression response to *Pou5f1 *and *Sox2 *suppression was similar when projected on the first principal component (PC1) (Fig 3C). PC1 seems to represent a transition of ES cells from a pluripotent state to more differentiated states because it is associated with a decreased expression of ES cell specific genes (e.g., *Nanog*, *Zfp42*, *Nr0b1*, *Tcl1*, *Dppa3*, *Klf4*, *Jarid2*), and increased expression of genes related to differentiation (e.g., *Esx1*, *Gata2*, *Gata6*, *H19*, *Hoxa1*, *Msx2*, *Plat*, keratins). The second principal component (PC2) represented minor fraction of genes that responded differentially to the suppression of *Sox2 *and *Pou5f1 *(Fig. 3D).

We did not have (Tet)-inducible *Nanog *ES cells, and thus had to use the following three experimental results: (i) time course (1 to 7 days) of shRNA-mediated knockdown in ES cells [5], (ii) shRNA-mediated knockdown of *Nanog *in ES cells [1], and (iii) stable over-expression of *Nanog *performed in our laboratory (see Materials and methods). The first data set was analyzed in a way similar to the *Pou5f1 *data set; however because the experiment was done without replications we combined data points (data from day 3 and 4, and data from day 5, 6, and 7) as replications for statistical analysis (Additional file 7). Combined data points showed similar gene expression profiles based on Principal Component Analysis (PCA) (data not shown). In the latter 2 data sets, we classified genes only based on expression change (positive vs. negative, and > 2 fold change vs. < 2 fold change), and used these classes for estimating the FDR (Additional files 8, 9). When we combined these 3 data sets, the direction of gene response was inverted for the experiment with *Nanog *over-expression to make it compatible with other data, and genes showing contradictory change were ignored. Because data on *Nanog *was obtained with different methods than on *Pou5f1 *and *Sox2*, it should be interpreted with caution.

It is important to note that genes affected by the alterations of *Pou5f1*, *Sox2*, and *Nanog *expression contain not only primary (direct) targets of these TFs, but also secondary/tertiary targets as shown previously [2].

### 2. Data set of genome-wide Chromatin IP

We used genome-wide ChIP data of POU5F1 and NANOG, published by Loh et al. [1]. All raw data were remapped to the mouse genome sequences and analyzed with the genome annotation using components of CisView software, which includes information on the transcription start sites (TSSs) [17]. As we pointed out in our previous work [2], some known POU5F1 target genes were missed in the POU5F1-target gene list assembled by Loh et al. [1] partly due to the use of a stringent criteria (i.e., ≥ 4 ditags). We therefore decided to use both POU5F1 and NANOG ChIP data, when we searched for POU5F1 target or NANOG target. Significant co-localization of POU5F1, SOX2, and NANOG binding sites have been clearly shown recently by ChIP-chip analysis [6]. In mouse, the strength of NANOG binding (measured by the number of ChIP-PET ditags) was positively associated with the strength of POU5F1 binding to the same region according to our re-analysis of ChIP data [1] (Additional file 10). Our analysis of TF binding motifs in DNA regions identified by ChIP [1] showed that ChIP-NANOG regions (isolated with NANOG antibody) had a high abundance of OCT-SOX composite binding motifs also known as HMG/POU cassettes [9,10] (Additional file 7). This finding also confirms co-localization of POU5F1, SOX2, and NANOG binding sites. Moreover, ChIP-NANOG regions that did not overlap with any ChIP-POU5F1 regions also had an increased abundance of OCT-SOX composite binding motifs (Additional file 7). This indicates that NANOG binding can be used as additional evidence of POU5F1 and SOX2 binding and vise versa. Therefore, it is reasonable to use a combination of POU5F1 and NANOG ChIP data for finding POU5F1 targets. For example, in the original ChIP-PET analysis by Loh et al., only binding sites with ≥ 4 ditags were considered reliable [1]. In contrast, in our modified approach we could utilize POU5F1 binding sites with only 2 or 3 ditags on condition that they had additional NANOG ditags. This approach increases the sensitivity of finding TF binding sites, but it may have a down side of possibly being too inclusive.

We also used genome-wide ChIP data for SOX2 obtained from human ES cells [18]. However, the human ChIP data were analyzed separately and used only to provide additional gene list for SOX2 targets (see the section 5 below for the details).

### 3. Evaluating the ChIP-binding sites with a score of potential function (SPF)

Presence of a ChIP-binding site of a TF in the promoter of a gene is not yet an evidence of transcription regulation because TF binding may be related to other cellular functions or may be not functional at all. To evaluate the potential functionality of POU5F1 binding sites in transcription regulation we developed a score of potential function (SPF), which was estimated using an ad hoc equation:

(1) SPF = [*N*_1_^*a *^+ (*b*·*N*_2_)^*a*^]·[max(*D*, 1000)/10000]^*c *^+ *d*·*X*,

where *N*_1 _and *N*_2 _are the number of ChIP-PET ditags for POU5F1 and NANOG (data from [1]), respectively, *D *is the distance from binding region to TSS, *X *= 1 for CpG-rich regions and 0 otherwise, and *a*, *b*, *c*, and *d *are adjustable parameters. As we discussed above, we used data on NANOG binding so that it can provide additional evidence of binding site function. Expression levels of *Nanog *changed > 48 hr after *Pou5f1 *suppression; thus, the training sets did not contain genes which responded to *Pou5f1 *suppression indirectly via the effect of *Nanog*, and the use of NANOG binding could not affect SPF in favor of indirect effects. The SPF was optimized to best separate between the training set of genes that responded to *Pou5f1 *suppression and control set of genes that were not affected by *Pou5f1*. We used 2 training sets of genes that were down-regulated (*N *= 782) and up-regulated (*N *= 519), respectively, by at least 2 fold and responded non-transiently to *Pou5f1 *suppression within the time window from 6 to 48 hr (Additional file 11). Genes that responded earlier than 6 hr may have been affected by other factors besides the gradually decreasing amount of POU5F1 protein, and genes that responded later than 48 hr are more likely to be affected indirectly. The control set of genes (*N *= 3048, Additional file 11) contained genes with medium- or high-quality promoters [17] represented by a responsive oligo in the microarray, which did not respond to *Pou5f1 *and had no differential expression between ES and TS cells [19]. Adjustable parameters were changed to maximize the *t*-statistics for the difference in average SPF values between the training and control set of genes (see Methods for details). To avoid circular reference by estimating SPF for genes in the training and control set with parameters optimized for the same genes, we used the bootstrap resampling method [20]. Scores for POU5F1 binding sites were positively affected by the number of ChIP ditags and negatively affected by the distance from TSS and by CpG richness of the sequence (Additional file 12). Down-regulated genes were more widely separated from control genes by their average SPF than up-regulated genes based on *t *statistics (Additional file 12). Characteristics of binding sites with highest SPF for each gene are given in Additional files 13 and 14.

### 4. Identification of Tentative Target Genes (TTGs) for POU5F1

We developed a novel algorithm to identify direct targets of a TF by first separating genes that responded to TF-manipulation into groups according to their expression patterns: direction (up-regulation vs. down-regulation), magnitude (> 2 fold vs. < 2 fold change) and time (in 12 hr intervals) of response. We then applied the FDR criterion to each group individually (Fig. 1). Genes with transient response were handled separately from genes with constitutive response. FDR was estimated in two steps: first *p*-values were estimated for each gene on the basis of the SPF of a binding site in the promoter and cumulative probability distribution of SPF for binding sites in promoters of control genes. The probability distribution of SPF in the control set of genes was approximated by a linear function log_*e*_(*p*|SPF > *x*) = *a *+ *b·x *(Additional file 15), and then the regression was used to calculate *p*-values. FDR was then calculated in each group of genes as described [21]. Numerical examples showing the advantage of this approach are given in the Methods section.

Assuming that we can tolerate up to 20% of false positives, we set FDR threshold to 0.2 and identified 420 TTGs of POU5F1 (Fig 2A, Additional file 4). The list of genes included the majority of known POU5F1 targets (*Sox2*, *Nanog*, *Zfp42*, *Klf4*, *Esrrb*, *Utf1*, *Lefty1*, *Otx2*, *Spp1*, *Upp1*, *Fbxo15*, *Dppa5*, *Cdyl*, *Cdx2*), which supports the validity of our analysis. Although one-fifths of these TTGs are in theory false positives due to FDR ≤ 0.2, we believe that this is the best result we can obtain with the current technology and uniform data analysis applied to all the genes. Some genes with strong response to *Pou5f1 *were not included in the list of TTGs (e.g., *Fgf4*), because the FDR values were slightly above the accepted threshold. We, therefore, assembled an additional list of 65 genes (Additional file 16) that are likely to be targets of POU5F1 although they did not pass our statistical criteria. These genes had either relatively low FDR values or additional evidence of their regulation by *Pou5f1*.

A list of target genes identified in this paper matched reasonably with lists of genes identified in earlier studies. Of 420 TTGs identified for *Pou5f1*, 82 genes overlapped with a list of genes identified in Matoba et al. [2], and 125 genes overlapped with a list of genes identified in Loh et al. [1] (Additional file 17). TTGs that were identified in earlier studies, but not identified in the current study fell into two categories: (i) TTGs that had weak binding sites; and (ii) TTGs that did not respond significantly to *Pou5f1 *manipulation in our experiment, as judged by the distribution of a simple score estimated as a product of SPF and absolute magnitude of gene expression response to *Pou5f1 *suppression (Additional file 18). The scores for TTGs identified in this paper were substantially higher than those for non-matching genes from the earlier papers, indicating that the current list of TTGs was high quality. Furthermore, there was significant overlap between TTGs identified in the current work and TTGs identified in the previous meta-analysis of gene expression in ES cells [22]: out of 83 genes with current gene symbols, 33 genes were identified as TTGs of POU5F1 in the current work.

**Figure 4:**
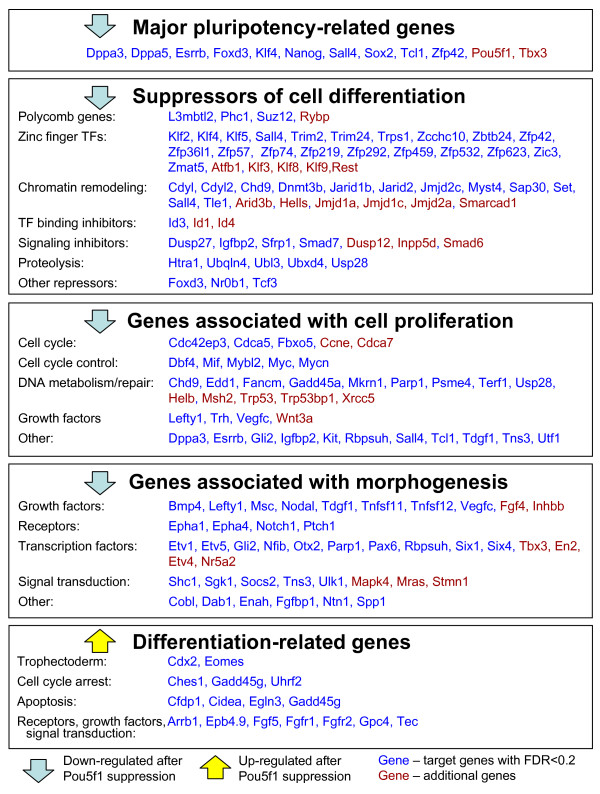
**Major functional groups of tentative target genes (TTGs) of POU5F1.** Genes in blue: genes that passed the criterion for TTGs (FDR < 0.2). Genes in red: additional genes that did not pass this criterion but are still likely to be targets.

Interestingly, we found that *Pou5f1 *functions mostly as a positive regulator of target gene expression in ES cells: among 420 TTGs of POU5F1, the majority (*N *= 372; 88.6%) were down-regulated after *Pou5f1 *suppression and only 48 TTGs were up-regulated (Fig. 2A). This was a surprise, because *Pou5f1 *is thought to suppress the expression of genes associated with cell differentiation. To address this issue, we analyzed 420 POU5F1-TTGs and 65 additional TTGs (from Additional file 16) based on Gene Ontology (GO) terms (Additional file 19) and literature (PubMed) and found 4 major categories for positive regulation and 1 category for negative regulation (Fig. 4). One notable category among genes activated by *Pou5f1 *was the "suppressors of cell differentiation," which was comprised of many known and hypothetical transcriptional repressors and signaling repressors (Fig. 4). These include polycomb genes such as *Suz12 *and *Phc1*, which are known to repress genes associated with differentiation in ES cells [23]. Zinc finger TFs are also known as suppressors of gene expression [24,25]. For example, *Klf4 *directly suppresses the expressions of *p53 *[26], *HDC *(*Hdc*) [27], and *Sp1 *[28], although *Klf4 *also activates a number of genes, including *Lefty1 *[29]. Some of zinc finger TFs activated by *Pou5f1 *(e.g., *Zfp57*, *Zfp74*, *Zfp459*) have KRAB domains, which are involved in gene-silencing and heterochromatin formation [30]. Some chromatin remodeling proteins are also known for suppressing gene expressions [31]. For example, *Dnmt3b *is a de novo DNA methyltransferase which can contribute to transcription silencing, although the gene seems dispensable for pluripotency maintenance [32]. *Cdyl *is a transcriptional co-repressor which is active via its CoA-pocket domain [33]. TFs with Jumonji domain (*JmjC*) function as histone demethylase and modulate the chromatin status [34]. Inhibitors of helix-loop-helix TF binding (*Id1*, *Id3*, *Id4*) are also included in the list. The "suppression of cell differentiation" category (Fig. 4) also includes a number of inhibitors of cell signaling, such as phosphatases (*Dusp27*, *Dusp12*, *Inpp5d*), inhibitors of TGFbeta signaling (*Smad7*), WNT inhibitor *Sfrp1*, and IGF inhibitor *Igfbp2*. Proteases are also included in the list: *Htra1 *inactivates TGFbeta signaling possibly via modification of ECM [35]; and ubiquitination-related genes (*Ubqln4*, *Ubl3*, *Ubxd4*, *Usp28*) may suppress gene activity via degradation of transcription-activation complexes. The *Pou5f1*, thus, seems to suppress the expression of genes associated with cell differentiation by positively regulating the "suppressors of cell differentiation."

Other groups of TTGs activated directly by POU5F1 are "major pluripotency-related genes", "genes associated with cell proliferation", and "genes associated with morphogenesis" (Fig. 4). The first group includes mostly known targets of POU5F1. Pluripotency-related genes, *Foxd3 *and *Sall4*, which are known for *Nanog *dependency [2,36], were also TTGs of POU5F1, because in our experiments they responded to *Pou5f1 *suppression long before the change of expression of *Nanog*. Germ line specific gene *Dppa3 *(*Stella, Pgc7*) was another new TTG of POU5F1. The group of "genes associated with cell proliferation" included major regulators of cell cycle (e.g., *Mif*, *Mybl2*, *Myc*, *Mycn*), DNA metabolism/repair genes, growth factors, and others, which are consistent with the earlier report [31]. *Mybl2 *(*b-Myb, Bmyb*) and *Myc *promote cell transition from G1 phase to S phase, and therefore contribute to the unique cell cycle structure (long S phase [37]) in ES cells [38,39]. The regulation of ES cell proliferation by *Tcl1 *has also been demonstrated previously [2]. Surprisingly, the group of "genes associated with morphogenesis", which included many embryonic growth factors, receptors, TFs, and signal transduction genes, appeared positively regulated by *Pou5f1*. Possibly, *Pou5f1 *is not the only transcription regulator of these genes because many of them remain active in developing embryo even after full silencing of *Pou5f1*.

In contrast, a small number of TTGs labeled as "differentiation-related genes" (e.g., *Cdx2 *and *Eomes*), was negatively regulated by *Pou5f1*. It has been shown that POU5F1 can block the transcriptional auto-activation of *Cdx2 *by CDX2 protein [36]. Although these genes have POU5F1 binding sites identified by ChIP, the functionality of these sites as transcription repressors has not been confirmed experimentally. If these binding sites are indeed involved in repression of transcription, then this mechanism may complement other more generic mechanisms of repression of genes associated with differentiation. For example, *Arrb1*, *Cdx2*, *Cidea*, *Eomes*, *Fgf5*, and *Gadd45g *have bivalent chromatin domains (carrying both activating H3K4 tri-methylation and repressing H3K27 tri-methylation) [40], and thus can be repressed via polycomb genes.

Thus, the main function of POU5F1 binding to promoters is activation of target genes, whereas suppression of genes related to cell differentiation is mostly indirect and mediated by specialized gene silencing mechanisms, many components of which are activated directly by *Pou5f1*. The *Pou5f1 *may have additional molecular functions besides direct regulation of transcription (e.g., inactivation of CDX2 protein via direct binding with POU5F1 protein in heterochromatin [36]); however these effects are beyond the scope of this paper. POU5F1 can interact directly with transcriptional repression complexes in ES cells [41], but it is not clear to what extent this interaction involves sequence-specific binding of POU5F1 to DNA.

### 5. Identification of target genes for SOX2 and NANOG

Target genes for SOX2 and NANOG were identified using the same strategy as for POU5F1 (Fig. 1). Data sources used for this analysis are summarized in Additional file 1. Many TTGs of SOX2 and NANOG overlapped with TTGs of POU5F1 (Additional file 20). Similarly to *Pou5f1*, *Sox2 *and *Nanog *seem to function as activators of gene expression rather than repressors: 222 genes (99.1%) were activated by *Sox2 *(out of 224 TTGs), and 251 genes (81.0%) were activated by *Nanog *(out of 310 TTGs) (Additional file 21; This table does not include genes that are regulated indirectly or via protein-protein interaction, e.g. by inactivation of SMAD1 by NANOG [42]).

We also assembled a table of additional TTGs of SOX2 based on human ES cell data, because ChIP data for SOX2 in mouse ES cells are not available and we might have missed important genes. We used ChIP data for SOX2 in human ES cells [6] and microarray data in mouse ES cells after *Sox2 *suppression (> 2 fold change of gene expressions) [16]. A list of genes is available in Additional file 22 for readers who are interested in these additional genes, but these genes were not used for further analyses described below. A gene list may contain a proportion of false positives larger than 20%, because the target genes of these TFs do not necessary overlap between human and mouse ES cells [43,1].

### 6. Common target genes of POU5F1, SOX2, and NANOG

**Figure 5:**
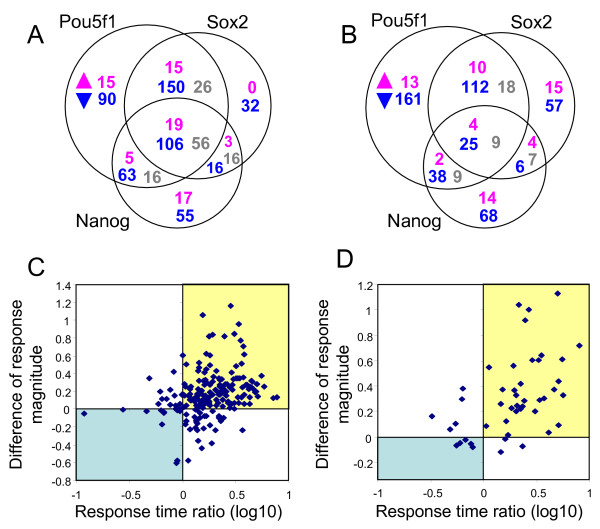
**Comparison of tentative target genes (TTGs) responding to the suppression of *Pou5f1*, *Sox2*, or *Nanog*.** (A) Venn diagram of combined TTGs of POU5F1, SOX2, and NANOG. Number of genes that were up-regulated (magenta), down-regulated (blue), or had a mixed response (gray) to the suppression of *Pou5f1*, *Sox2*, and *Nanog*. (B) The same Venn diagram as (A) after removing genes with possible indirect effects. (C) Comparison of gene responses to the suppression of *Pou5f1 *and *Sox2 *(TTGs of POU5F1 that were down-regulated after *Pou5f1 *suppression): response time ratio = log10(*t*_2_/*t*_1_) where *t*_1 _and *t*_2 _are response times to *Pou5f1 *and *Sox2 *suppression, respectively; difference of response magnitude = *m*_2 _- *m*_1_, where *m*_1 _and *m*_2 _are response magnitudes (log-ratios) to *Pou5f1 *and *Sox2 *suppression, respectively; genes in the blue quadrant responded more strongly and faster to *Sox2 *suppression than to *Pou5f1 *suppression, and genes in the yellow quadrant responded more strongly and faster to *Pou5f1 *suppression than to *Sox2 *suppression. (D) Comparison of gene responses to the suppression of *Pou5f1 *and *Nanog *(TTGs of *Pou5f1 *that were down-regulated after *Pou5f1 *suppression); notations are the same as in (C).

It has been shown that POU5F1, SOX2, and NANOG proteins co-occupy promoters of a substantial portion of their target genes in the analysis of ChIP-Chip data of human ES cells [6]. Consistent with this notion, new lists of TTGs identified in this paper based on both ChIP data and expression profiling data showed that out of 700 combined TTGs of POU5F1, SOX2, and NANOG, only 209 genes (29.9%) were affected by one of these TFs; other TTGs were affected by 2 or 3 TFs (Fig. 5A, Additional file 21). The majority of TTGs were down-regulated after suppression of *Pou5f1*, *Sox2*, and/or *Nanog *(*N *= 512, 73.1%), very few were upregulated (*N *= 74, 10.6%), and some had a mixed response (*N *= 114, 16.3%) (Fig. 5A).

One of the concerns was that these common TTGs could have been erroneously identified, because *Pou5f1*, *Sox2*, and *Nanog *affect the expression of each other and thereby indirectly affects downstream gene expressions. To eliminate possible indirect effects, we used our observation that manipulation of each of these 3 genes changed the expression of other genes with a substantial delay (> 34 hr), which makes it possible to separate gene networks affected by each TF. We plotted another Venn diagram where effects of *Pou5f1*, *Sox2*, and *Nanog *were counted only if target genes responded before the response time of other members within this group of 3 major TFs associated with ES cell pluripotency (Fig. 5B). Even after removal of these possible indirect effects, correlation between effects of *Pou5f1*, *Sox2*, and *Nanog *remained strong.

We also examined a possibility that these co-regulations occurred by chance. For example, the number of genes activated by both *Pou5f1 *and *Sox2 *(*N *= 137, Fig. 5B) was significantly higher than expected from permutation of 1729 genes with strong binding sites (with SPF > 95-percentile in control genes); expected overlap was only 43.61 genes (chi-square = 50.95, *p *< 0.001). Permutation was limited to genes with strong binding sites to avoid the possibility that apparent correlation between effects of *Pou5f1*, *Sox2*, and *Nanog *simply resulted from the use of the same ChIP dataset for selecting TTGs for these TFs. The number of genes activated by both *Pou5f1 *and *Nanog *(*N *= 63) was also significantly higher than expected from permutation (31.96 genes; chi-square = 10.43, *p *= 0.001). In contrast, co-activation of genes by *Sox2 *and *Nanog *appeared non-significant: only 31 genes were activated by both TFs versus expected 17.99 genes (chi-square = 3.51, *p *= 0.06). Effect of *Pou5f1 *and *Sox2 *was similar not only in activation but also in suppression of target genes. The number of genes suppressed by both *Pou5f1 *and *Sox2 *(*N *= 14) was statistically higher than expected from permutation (expected *N *= 1.23, chi-square = 10.75, *p *= 0.001). These genes included cell-cycle suppressor *Gadd45g*, FGF-signaling genes (*Fgf5*, *Fgfr2*), and TFs *Eomes *and *Dmrt1*.

Expression of TTGs of POU5F1, SOX2, and NANOG may be regulated by other factors as well. To distinguish genes that are predominantly regulated by *Pou5f1 *and *Nanog*, we analyzed the expression of TTGs in lineage committed cell types reported previously: mouse trophoblast stem cells (TS), neural stem cells (NS), and embryonic fibroblasts (MEF) [44,45,19]. *Pou5f1 *and *Nanog *are not expressed in these cell types. However, *Sox2 *is expressed in NS and TS (lower expression levels than in ES cells), but is not expressed in MEF. We expected that genes that are predominantly regulated by *Pou5f1 *and *Nanog *will change their expressions in these cell types in the same direction as in the ES with suppressed expression of *Pou5f1 *or *Nanog*. Out of 700 TTGs, 383 were consistently down-regulated and 94 were consistently up-regulated in lineage-committed cells (Additional file 21). In the former group 92.7% genes were down-regulated after suppression of either *Pou5f1 *(*N *= 324) or *Nanog *(*N *= 159), and in the latter group only 42.6% genes were up-regulated after suppression of *Pou5f1 *(*N *= 33) or *Nanog *(*N *= 18). In total, we found 395 TTGs that were expected to be predominantly regulated by *Pou5f1 *and *Nanog*, and the majority of them (*N *= 355) were activated by these TFs (Additional file 21).

### 7. Differential effects of *Pou5f1*, *Sox2 *and *Nanog *on their TTGs

Although *Pou5f1*, *Sox2*, and *Nanog *cooperate strongly in their effects on target genes, their roles may not be equal. For example, *Sox2 *is dispensable in activation of several known gene expression enhancers with OCT-SOX composite binding site [16]. Thus, we wanted to check if the same was true for other genes activated by *Pou5f1*. Among 378 TTGs of POU5F1 that were down-regulated after *Pou5f1 *suppression, 131 were not affected by *Sox2 *suppression, and 148 responded more weakly and with delay compared to their response to *Pou5f1 *suppression (Fig. 5C, upper right quadrant). Some genes from the latter set may be affected by *Sox2 *only indirectly via *Pou5f1 *suppression; however testing this hypothesis would require further study. Only 12 genes responded to *Sox2 *suppression more strongly and faster compared with their response to *Pou5f1 *suppression (Fig. 5C, lower left quadrant).

Similarly, out of 378 TTGs of POU5F1 that were down-regulated after *Pou5f1 *suppression, 237 were not affected by *Nanog*, and 33 responded to *Nanog *suppression weaker and with delay compared to their response to *Pou5f1 *suppression (Fig. 5D, upper right quadrant). Small number of genes responding to *Nanog *manipulation can be partially explained by the fact that suppression of *Nanog *did not change the expression of *Pou5f1 *in both knockdown and overexpression experiments, and therefore, there were no indirect effects mediated by *Pou5f1*. Only 5 genes responded to *Nanog *suppression more strongly and faster compared to their response to *Pou5f1 *suppression (Fig. 5D, lower left quadrant). Genes that responded more strongly (but not necessary faster) to *Nanog *suppression compared to *Pou5f1 *suppression (*N *= 13) were enriched in genes associated with growth factor activity (*Bmp4*, *Spred2*, *Spry4*, *Igfbp2*). Thus, although *Pou5f1*, *Sox2*, and *Nanog *cooperate in activation of TTGs, *Pou5f1 *plays the major role in this cooperation, whereas *Sox2 *and *Nanog *generally have a weaker effect. However, there were exceptions from this general rule because some genes were affected more strongly by *Sox2 *or *Nanog *than by *Pou5f1*.

**Figure 6:**
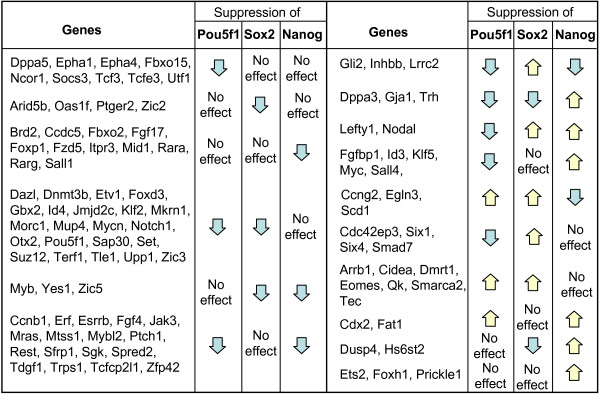
Examples of tentative target genes responding differentially to the suppression of *Pou5f1*, *Sox2*, and *Nanog*.

Examples of differential effects of *Pou5f1*, *Sox2*, or *Nanog *on their TTGs are given in Fig. 6. Many suppressors of cell differentiation (e.g., *Dnmt3b*, *Foxd3*, *Id4*, *Jmjd2c*, and *Suz12*) were activated by *Pou5f1 *and *Sox2 *but not by *Nanog*, whereas several genes associated with ES cell pluripotency (*Esrrb*, *Sfrp1*, *Tdgf1*, *Zfp42*) were activated by *Pou5f1 *and *Nanog *but not by *Sox2*. Some pluripotency-related genes (e.g., *Dppa5 (Esg1) *and *Utf1*) were activated solely by *Pou5f1*. Germline markers *Dppa3 *(*Stella*) and *Dazl *were activated by *Pou5f1 *and *Sox2 *but either suppressed (*Dppa3*) or not affected (*Dazl*) by *Nanog*. Trophectoderm marker *Cdx2 *was suppressed by *Pou5f1 *and *Nanog*, whereas another trophectoderm marker *Eomes *was suppressed by *Pou5f1 *and *Sox2 *but not by *Nanog*. Genes associated with morphogenesis, *Lefty1 *and *Nodal*, were suppressed by *Sox2 *and *Nanog *but activated by *Pou5f1*. Differential effect of *Pou5f1*, *Sox2*, and *Nanog *on target genes may be important for embryo patterning and regulation of metabolism.

Some differential effects were observed on quantitative level only. For example, TTGs of *Sox2 *that were down-regulated more strongly (but not necessary faster) after *Sox2 *suppression compared to *Pou5f1 *suppression (*N *= 100, Additional file 21) were enriched in genes associated with neural differentiation (*Dab1*, *Fut9*, *Notch1*, *Nrp2*, *Pax6*, *Gbx2*, *Nef*, *Zic2*, *Zic5*), and first 5 of these genes were over-expressed in NS compared to ES, according to published data [45,44]. Interestingly, additional TTGs of SOX2 that had no POU5F1 or NANOG binding sites, were also enriched in genes that were over-expressed in NS (e.g., *Cdh2*, *Dpysl3*, *Fez1*, *Lrrn1*, *Pdzrn4*, *Sema6a*, *Timp4*, *Vim*, *Zic1*) (Additional file 22). These observations are consistent with a known role of *Sox2 *in neural differentiation [46,47].

## Conclusion

We have developed a novel algorithm to evaluate the statistical significance of direct downstream target genes for a TF. The method uses two data inputs: genome-wide time-course expression profiling data after the manipulation of TF expression level; and genome-wide ChIP data. We have applied this method to key pluripotency genes – *Pou5f1*, *Sox2*, and *Nanog*, and identified their TTGs in ES cells. Because the majority of TTGs were activated and only a few of them were suppressed, we conclude for the first time that the main function of *Pou5f1*, *Sox2*, and *Nanog *when they are bound to promoters is activation of gene expression rather than suppression. Thus, the role of these TFs in suppression of genes associated with differentiation is mostly indirect and is mediated via specialized gene silencing mechanisms. This idea is supported by our observation that transcription of many components of these silencing pathways is directly activated by *Pou5f1*, *Sox2*, and *Nanog*. These 3 genes may have additional molecular functions that are not related to binding to the promoters of target genes, but these functions cannot be inferred from ChIP data and therefore require different methods of analysis.

## Methods

### Microarray experiments

For microarray experiments we used ZHBTc4 ES cells with a Tet-inducible *Pou5f1 *transgene [4]. Cells were cultured for 2 passages on gelatin-coated plates in order to remove feeder cells and then transferred to gelatin-coated 6-well plates at the density of 1–2 × 10^5 ^cells/well and cultured in complete ES medium: DMEM, 15% FBS; LIF (ESGRO, Chemicon, USA) 1000 U/ml; 1 mM sodium pyruvate; 0.1 mM NEAA, 2 mM glutamate, 0.1 mM beta-mercaptoethanol, and penicillin/streptomycin (50 U/50 μg per ml). Tetracycline was added at 24 hr after cell plating, and then cells were harvested at 0 hr (before adding tetracycline), 3 hr, 6 hr, 12 hr, and 24 hr (2 replications each). RNA samples for later time points (24, 48, 72, 96, and 120 hr) were obtained from our earlier experiment with 3 replications [2]. Two *Nanog *over-expressing clones were tested: integrated and episomal transgene. To generate an integrated transgene, *Nanog *cDNA was amplified by PCR and cloned into pEF6/pIRESneo3 vector (Invitrogen). *Nanog*-pEF6/pIRESneo3 construct was transfected into MC1 cell line (129S6/SvEvTac strain; purchased from the Transgenic Core Laboratory of the Johns Hopkins University School of Medicine). After 10 days of selection in 300 ug/ml G418 (Invitrogen), resistant colonies were picked and expanded. To generate an episomal transgene, plasmid construct was made by cloning *Nanog *cDNA into pPyCAGIP episomal expression vector [48]. *Nanog*-pPyCAGIP episomal expression construct was transfected into MG1.19 cell line, expressing polyoma T large antigen [49]. After 7 days of puromycin selection, 2 μg/ml, resistant colonies were picked, expanded and *Nanog *expression level was confirmed by real time PCR: it was 2.14 ± 0.14 fold for the integrated clone and 19.6 ± 1.1 fold for the episomal clone. Both *Nanog *transgenic and parental cell lines were cultured for 2 passages on gelatin-coated plates and then transferred to gelatin-coated 6-well plates at the density of 1–2 × 10^5 ^cells/well and cultured for 3 days in 3 different conditions: (1) complete ES medium (see above); (2) complete medium without LIF, and (3) complete medium with 1 μM RA. Cells were cultured at 37°C and 5% CO^2 ^condition and the culture medium was changed daily.

Total RNAs were extracted using Trizol™ (1 ml/well; Invitrogen, USA) and Phase lock gel™ columns (Eppendorf/Brinkman) according to the manufacturer's protocol. Total RNAs were precipitated with isopropanol, washed with 70% ethanol, and dissolved in DEPC-treated H2O. 2.5 μg of total RNA samples were labeled with Cy3-CTP using a Low RNA Input Fluorescent Linear Amplification Kit (Agilent, USA). A reference target (Cy5-CTP-labeled) was prepared from the Universal Mouse Reference (UMR) RNA (Stratagene, USA). Labeled targets were purified using an RNeasy Mini Kit (Qiagen, USA) according to the Agilent's protocol, quantified by a NanoDrop scanning spectrophotometer (NanoDrop Technologies, USA), and hybridized to the NIA Mouse 44 K Microarray v2.1 (whole genome 60-mer oligo; manufactured by Agilent Technologies, #012799) and NIA Mouse 44 K Microarray v2.2 (whole genome 60-mer oligo; manufactured by Agilent Technologies, #014117) [19] according to the Agilent protocol (G4140-90030; Agilent 60-mer oligo microarray processing protocol – SSC Wash, v1.0). RNA samples from experiment of [2] were hybridized to NIA Mouse 22 K Microarray Dev2 (Agilent Technologies, design #012165) [19]. All hybridizations were carried out in the two color protocol by combining one Cy3-CTP-labeled experimental target and Cy5-CTP-labeled reference target. Microarrays were scanned on an Agilent DNA Microarray Scanner, using standard settings, including automatic PMT adjustment.

### Statistical analysis of microarrays

The data discussed in this publication have been deposited in NCBI Gene Expression Omnibus and are accessible through GEO Series accession number (GSE8617). All the microarray data are available at the public GEO website [50,51]. The data and analysis software are also available at the NIA Array Analysis website [52,53]. Because some arrays showed slight reduction of Cy5 signal (UMR) due to ozone bleaching we compensated for this effect as follows. First we selected 100 genes with the highest variances of Cy5 signal and with average log10-signal > 2.5, and estimated the average Cy5 signal for these genes in each array which roughly represented the degree of bleaching effect. Then we used a linear regression to fit Cy5 log signal for each gene as a function of the bleaching effect (average log Cy5 signal of 100 genes described above), and the Cy3 signal in the same array. Then the effect of bleaching was subtracted. If after this correction, log Cy5 signal in array differed by > 3·SD from the mean log Cy5 signal it was replaced by the mean. Data on earlier time points (3–24 hr) was combined with data on later time points (24–120 hr)[2] that was adjusted as follows: *x*'_*t *_= *x*_*t *_- *x*_24 _+ *y*_24_, where *x*_*t *_and *x*'_*t *_are log gene expression for time point > 24 hr before and after adjustment, respectively, *x*_24 _is log gene expression in the experiment of [2], and *y*_24 _is log gene expression in the new experiment. For the majority of genes we used the same oligo sequence in both data sets that were combined. However, for some genes that had no common oligo we combined data from different oligos. Data on genes that had no oligo (or no sensitive oligo) in NIA Mouse 22 K Microarray Dev2 (*N *= 109) were taken from NIA Mouse 22 K Microarray Dev1 presented in [2]. Information on *Cdx2 *is based on PCR data (Additional file 23) because our microarray probe for this gene was not responsive. Source of data for each gene is listed in Additional file 2.

Statistical analysis was done using the NIA Array Analysis software [52]. To reduce the number of false positives we used the maximum of the actual error variances for a gene and the average error variance estimated from 500 genes with similar signal intensity. Difference in expression was considered significant on the basis of false discovery rate FDR ≤ 0.05, which accounts for the effect of multiple hypotheses testing. Time of gene response to *Pou5f1 *suppression was estimated as the time when interpolated gene expression reached the level of 1.5 fold difference compared to the 0 hr initial time point, and the magnitude of response was estimated as the maximum log-ratio of gene expression (either positive or negative) compared to 0 hr (see Fig. 2C). Expression change was considered transient if after reaching its peak (positive or negative), it declined to < 1.5 fold change or became inverted. Gene ontology (GO) analysis was done using the NIA Mouse Gene Index software, which evaluates statistical significance using the hypergeometric probability distribution with parameters: FDR = 0.05, over-representation ratio > 1.5 fold [54-56].

### Identification of tentative target genes (TTGs)

To identify TTGs we optimized parameters of the score of potential function (SPF) (equation 1) to distinguish best between training and control sets of genes. Training sets were genes that were down-regulated or up-regulated by at least 2 fold and responded non-transiently to *Pou5f1 *suppression within the time window from 6 to 48 hr (Additional file 11). Genes that responded too early (< 6 hr) were not included in the training set because there may have been not enough time for the concentration of *Pou5f1 *protein to drop substantially (see Additional file 3), and therefore the response may have been caused by other factors. Similarly genes that responded late (> 48) were not included in the training set because many of them may be affected indirectly. The set of control genes was defined as a set of genes with high-quality or medium-quality promoters [17]which did not respond to suppression of *Pou5f1 *in ZHBTc4 ES cells (FDR > 0.05) and were not differentially expressed between ES and TS cells (FDR > 0.05) [19]. *Pou5f1 *gene is expressed in ES cells but not in TS cells [57], therefore we expected that the expression of *Pou5f1 *target genes was different in these types of cells. No change in gene expression measured by microarray may have resulted from non-responding oligos. Thus, we further narrowed down the list of control genes to those genes that showed differential expression (FDR ≤ 0.05 and > 1.5 fold change) between ovary and testis measured with the same array platform [11] to confirm the functionality of oligos. SPF was estimated based on the strength of *Pou5f1 *and *Nanog *binding [1], distance from TSS, and CpG-richness of the DNA region. TSS coordinates in mouse genome assembly mm6 were taken from [17], and coordinates of ChIP-PET regions were converted to genome version mm6 using UCSC batch conversion tool [58]. Association between binding sites and TSSs was established using the following rules: (1) if distance to TSS of gene A was > 3 fold greater than distance to TSS of gene B, then binding site was associated only with gene B, otherwise it was associated with both A and B; (2) if genes A and B have a common bidirectional promoter with distance between TSSs < 1 K then binding sites were associated with both genes; (3) non-RefSeq genes were ignored unless they responded to *Pou5f1 *manipulation (this rule was needed to avoid association with antisense regulatory transcripts); and (4) distance between binding site and TSS was limited to 200 K. CpG-rich regions were those that contained minimum of 8 CpG pairs within 250 bp [17]. Initially we included the effect of repeats and binding motifs in SPF, but then we removed them because they were not significant. Optimization was done separately for 2 training sets of genes that were down-regulated and up-regulated after suppression of *Pou5f1*. To optimize the parameters of the SPF we used the simplex method implemented in a Perl script based on the published algorithm [59]. At each step of optimization, scores were re-calculated according to modified parameters, binding sites with the highest score were selected for each gene, and then these scores were compared between training genes and control genes using *t*-statistics. To avoid a circular reference by estimating SPF for genes in the training and control set with parameters optimized for the same genes, we used the bootstrap resampling method [20]. Both training and control sets of genes were randomly split into 10 portions, and optimization was repeated 10 times with one portion of training and control genes excluded. Then, parameters (*a*, *b*, *c*, and *d*) in equation (1) were averaged; however when estimating SPFs for genes that belong to the *i*-th portion of training or control genes we averaged only those values that were optimized without the *i*-th portion of genes. The probability distribution of binding scores among control genes was used for estimating the *p*-value, and then the FDR was estimated using the method of [21] in each group of genes that differ by time, direction, and magnitude of response to manipulation of the TF. TTGs were selected using FDR = 0.2 as a threshold. Because each group has a large number of genes (median = 160), FDR in each group can be interpreted as the proportion of false positives among genes that are assumed significant in this group. Thus, the total proportion of false positives among pooled significant genes from all groups should also be close to the FDR threshold. This method is based on the assumption that SPF represents correctly the role of binding sites in transcription regulation. This assumption is not always true because SPF estimation is based on the limited information that is available; thus binding sites with the highest SPF for each gene are not necessary the ones that act as main regulatory switches. The quality of analysis can be improved as additional information becomes available (e.g., luciferase assay and location of insulators).

### Numerical examples

The purpose of these examples is to show the advantage of the proposed method for detecting direct target genes compared to the traditional approach when all genes that responded to manipulation of a TF and have a binding site in their promoter are considered direct targets. Our method for identification of direct targets of a TF is based on splitting the genes that responded to TF manipulation into groups according to the direction, magnitude and time of their response, and then applying the FDR criterion within each group (Fig. 1). In the first example we assume that all true binding sites are known, and therefore there is no need for using SPF. There are 5000 genes that responded to manipulation of TF which are split into 10 groups according to the direction, magnitude and time of response. For simplicity we assume that all groups have equal number of genes, and only 2 of these groups contained true direct target genes. The proportion of target genes in these 2 groups was 40% and in other groups it was 0%. Binding sites are present in promoters of all true target genes and in 20% of other genes (non-functional binding). If we consider all responding genes with binding sites as TTGs, then we would find 1320 of these genes which include 69.7% of false positives. Using our approach, we select genes with binding sites only in 2 groups of responding genes that had a significantly higher proportion of genes with binding sites (52% versus 20% in control). As a result, 520 TTGs are detected and they contain only 23% of false positives. Here our method yielded a 3-fold reduction in the proportion of false positives. The second example, which is more realistic, assumes that there is no clear-cut distinction between true and false binding sites. Instead, binding sites are characterized quantitatively by SPF. We modify the previous example by assuming that true target genes have a normal distribution of SPF with M = 7 and SD = 2, whereas other genes have a normal distribution of SPF with M = 3 and SD = 2. Assuming that we can tolerate 25% of false positives, we set FDR threshold to 0.25. Using the method of [21] and p-values from the probability distribution of SPF in control genes, we estimated that this FDR = 0.25 corresponds to the threshold of SPF = 5.75 in groups that contain true target genes, and is unattainable in groups without true target genes. As a result, 347 TTGs are identified, and 52 of them are false positives (15%). Proposed method reduced the proportion of false positives by > 3 fold and increased the number of identified true target genes by 47%. The actual proportion of false positives appeared slightly lower than the target FDR rate because we assumed that the distribution of SPF among true targets is unknown.

### Real-time quantitative RT-PCR

Primers for quantitative reverse transcriptase PCR (qRT-PCR) were designed and tested for SYBR Green chemistry using an established in-house protocol [57]. Primers for *Cdx2 *were ACGTACATGGTGGCGAGGGA (forward), and GGGAGGCAGAAGCTCTGCAA (reverse), and primers for *Nanog *were CTGGGAACGCCTCATCAA (forward), and CATCTTCTGCTTCCTGGCAA (reverse). Primers for *Pou5f1 *(transgene-specific) were ACGAGTGGAAAGCAACTCA and AGATGGTGGTCTGGCTGAAC. Total RNA was used to prepare cDNA as described previously [57]. Reactions were run on ABI 7900 HT Sequence Detection Systems using the default cycling program, and data were processed using SDS 2.2 software (Applied Biosystems).

### Western blot analysis

Protein amounts of each sample were quantified using the RC DC protein assay kit (Bio-Rad, 500–0119). For western blotting, the samples were boiled for 5 min and loaded onto a 8–16% SDS-polyacrylamide gel. The electrophoretically separated proteins were transferred to immobilon-P membrane (Millipore). The membrane filters were blocked with PBST (1× PBS, 0.05% Tween 20) containing 5% nonfat milk powder (PBST-Milk) at room temperature for 1 h and then washed three times each with PBS-T for 5 min. The primary antibodies against POU5F1 (sc-5279, Santa Cruz Biotechnology, Inc.) and UBTF (UBF) (sc-13125, Santa Cruz Biotechnology, Inc.) diluted at 1:10000 in PBST-Milk were applied at room temperature for 1 h. After three washes with the PBS-T, the secondary HRP-conjugated IgG (1:3000 dilution) in PBST-Milk was applied at room temperature with agitation for 30 minutes. The filters were washed again, treated with the ECL Western Blotting Detection Reagents (GE Healthcare, USA), and exposed to a film for visualization.

## Abbreviations

ChIP, chromatin-immunoprecipitation; ES, embryonic stem (cells); FDR, false discovery rate; GO, gene ontology; MEF, mouse embryonic fibroblast; NS, neural stem (cells); PCA, principal component analysis; SPF, score of potential function (for binding sites); TF, transcription factor; TS, trophoblast stem (cells); TSS, transcription start site; TTG, tentative target gene.

## Authors' contributions

AAS developed the method for identification of target genes of transcription factors, carried out statistical and bioinformatics analysis, and wrote the paper. SM carried out the experiment on suppression of *Pou5f1 *and *Sox2 *expression in ES cells. LVS carried out the experiment on *Nanog *over-expression and did PCR. YP labeled and hybridized probes to microarrays. KA and RM carried out the experiment on suppression of *Pou5f1 *expression in ES cells. LX performed Western blot for POU5F1. HN developed inducible knockout cell lines. MSHK coordinated the project and edited the manuscript. All authors read and approved the final manuscript.

## Supplementary Material

Additional file 1 Summary of data used in this study Click here for file

Additional file 2 **Log-transformed gene expression data for non-redundant genes in ZHBTc4 cell line after suppression of *Pou5f1***. The time series was combined from 2 data sets: (1) data for 0, 3, 6, 12, and 24 hr after adding tetracycline analyzed with NIA 44 k array (probe names in the 1st column), and (2) data for 0, 24, 48, 72, 96, and 120 hr are from the experiment of [2] analyzed with NIA 22 k Dev-2 array (probe names are in column 24). Click here for file

Additional file 3 **Suppression of tet-inducible transgene *Pou5f1 *in ZHBTc4 ES cells in a time course after adding tetracycline to the media**. (A) Log expression change from microarray data (log10), oligo is in ORF. (B) Expression change in real-time PCR data normalized by expression in parental EB5 cell line, primers are in transgene-specific region. (C) Western blot showing decrease in POU5F1 protein amount, UBTF is used as a control. Click here for file

Additional file 4 Genes that responded to *Pou5f1 *suppression in ZHBTc4 ES cells Click here for file

Additional file 5 **Comparison of genes that responded to *Pou5f1 *suppression in various studies**. (A) Venn diagram of genes that responded to *Pou5f1 *suppression by > 2 fold in our experiment with tet-inducible ES cell line ZHBTc4 and in published studies of [5,1], which used shRNA as a method of *Pou5f1 *suppression. (B) Scatter-plot of gene expression change after *Pou5f1 *suppression in this study and in [1] based on 1225 common genes. Click here for file

Additional file 6 Genes that responded to *Sox2 *suppression in 2TS22C ES cells (data from [16]) Click here for file

Additional file 7 Genes that responded to *Nanog *suppression with shRNA in ES cells (analysis of data from [5]) Click here for file

Additional file 8 Genes that responded to *Nanog *suppression with shRNA in ES cells (analysis of data from [1]) Click here for file

Additional file 9 Genes that responded to *Nanog *overexpression in ES cells Click here for file

Additional file 10 **Co-localization of POU5F1 and NANOG binding sites in the mouse genome based on ChIP-PET data from **[1]. (A) Proportion of ChIP-POU5F1 regions with different number of ditags that were co-localized (withn 1 Kb) with ChIP-NANOG regions (number of ditags indicates binding strength); (B) Density distribution of OCT-SOX composite binding motifs at various distances from the binding site identified with ChIP-PET [1] estimated for groups of genes with ≥ 4 POU5F1 ditags, with ≥ 4 NANOG ditags, and with ≥ 4 NANOG ditags and no POU5F1 ditags. Search for binding motifs was done using components of CisView software [17], which combines pattern-matching and position-weight matrix (PWM) approaches. PWM for OCT-SOX composite binding site was taken from [1]. Click here for file

Additional file 11 Sets of training genes that responded to *Pou5f1 *suppression and control genes used for optimizing the score of potential functionality of binding sites Click here for file

Additional file 12 Parameters of equation for the score of potential functionality (SPF) Click here for file

Additional file 13 POU5F1 binding sites with the highest score of potential functionality (SPF) for each gene Click here for file

Additional file 14 ChIP-PET regions (from [1]) listed in Additional file 8 Click here for file

Additional file 15 **Regression of the cumulative proportion of control genes (p) versus the score of potential function (SPF) of their binding regions identified with ChIP-PET **[1]. Cumulative proportion of genes was estimated after sorting them by decreasing SPF. Magenta line shows the regression estimated using top 300 genes with highest SPF. (A) SPF parameters estimated by optimization against down-regulated genes; (B) SPF parameters estimated by optimization against up-regulated genes. Click here for file

Additional file 16 Additional tentative target genes for POU5F1. Click here for file

Additional file 17 Comparison of tentative target genes of POU5F1 identified in this paper with previously published lists of genes Click here for file

Additional file 18 Frequency distribution of a score [SPF·abs(logratio)] among tentative target genes TTGs of POU5F1 identified in this paper and TTGs (non-matching with ours) from [1,2] Click here for file

Additional file 19 Gene Ontology (GO) annotations of POU5F1 tentative target genes Click here for file

Additional file 20 Venn diagram of tentative target genes (TTGs) of POU5F1, SOX2, and NANOG Click here for file

Additional file 21 Combined tentative target genes (TTGs) of POU5F1, SOX2, and NANOG Click here for file

Additional file 22 Additional tentative target genes for SOX2 identified using ChIP data from human ES cells [6] and > 2 fold expression change in mouse ES cells [16] Click here for file

Additional file 23 Expression change of *Cdx2 *after suppression of *Pou5f1 *and *Sox2 *analyzed using quantitative RT-PCR (SE estimated with ANOVA) Click here for file
