# Supplementary material for: Identification of Pou5f1, Sox2, and Nanog downstream target genes with statistical confidence by applying a novel algorithm to time course microarray and genome-wide chromatin immunoprecipitation data
Source: BMC Genomics. 2008 Jun 3;9:269. doi: 10.1186/1471-2164-9-269 (PMC2424064; doi:10.1186/1471-2164-9-269)
Supplement: Additional file 1 — Summary of data used in this study [file 1471-2164-9-269-S1.pdf]

Transcription factors	Genome-wide gene expression profiles	Genome-wide chromatin immunoprecipitation (ChIP) assays
Pou5f1 (Oct3/4, Oct4)	• Repression of Pou5f1 by tetracycline-controllable Pou5f1 in ZHBTc4 ES cells (mouse: Matoba et al., 2007)<br>• Repression of Pou5f1 by tetracycline-controllable Pou5f1 in ZHBTc4 ES cells (mouse: new data for 0, 3, 6, 12, 24 hrs)	• ChIP-PET for Pou5f1 (mouse: Loh et al., 2006)<br>• ChIP-PET for Nanog (mouse: Loh et al., 2006)
Nanog	• Repression of Nanog by shRNA (mouse: Ivanova et al., 2006)<br>• Repression of Nanog by shRNA (mouse: Loh et al., 2006)<br>• Overexpression of Nanog by a Nanog-transgene integrated into the genome in ES cells (mouse: new data)<br>• Overexpression of Nanog by an episomal vector in ES cells (mouse: new data)	• ChIP-PET for Pou5f1 (mouse: Loh et al., 2006)<br>• ChIP-PET for Nanog (mouse: Loh et al., 2006)
Sox2	• Repression of Sox2 by tetracycline-controllable Sox2 (mouse: Masui et al., 2007)	• ChIP-PET for Pou5f1 (mouse: Loh et al., 2006)<br>• ChIP-PET for Nanog (mouse: Loh et al., 2006)<br>• ChIP-chip for Sox2 (human: Boyer et al., 2005)

Additional file 1. Summary of data used in this study
