# Supplementary material for: Identification of Pou5f1, Sox2, and Nanog downstream target genes with statistical confidence by applying a novel algorithm to time course microarray and genome-wide chromatin immunoprecipitation data
Source: BMC Genomics. 2008 Jun 3;9:269. doi: 10.1186/1471-2164-9-269 (PMC2424064; doi:10.1186/1471-2164-9-269)
Supplement: Additional file 3 — Suppression of tet-inducible transgene Pou5f1 in ZHBTc4 ES cells in a time course after adding tetracycline to the media. (A) Log expression change from microarray data (log10), oligo is in ORF. (B) Expression change in real-time PCR data normalized by expression in parental EB5 cell line, primers are in transgene-specific region. (C) Western blot showing decrease in POU5F1 protein amount, UBTF is used as a control. [file 1471-2164-9-269-S3.pdf]

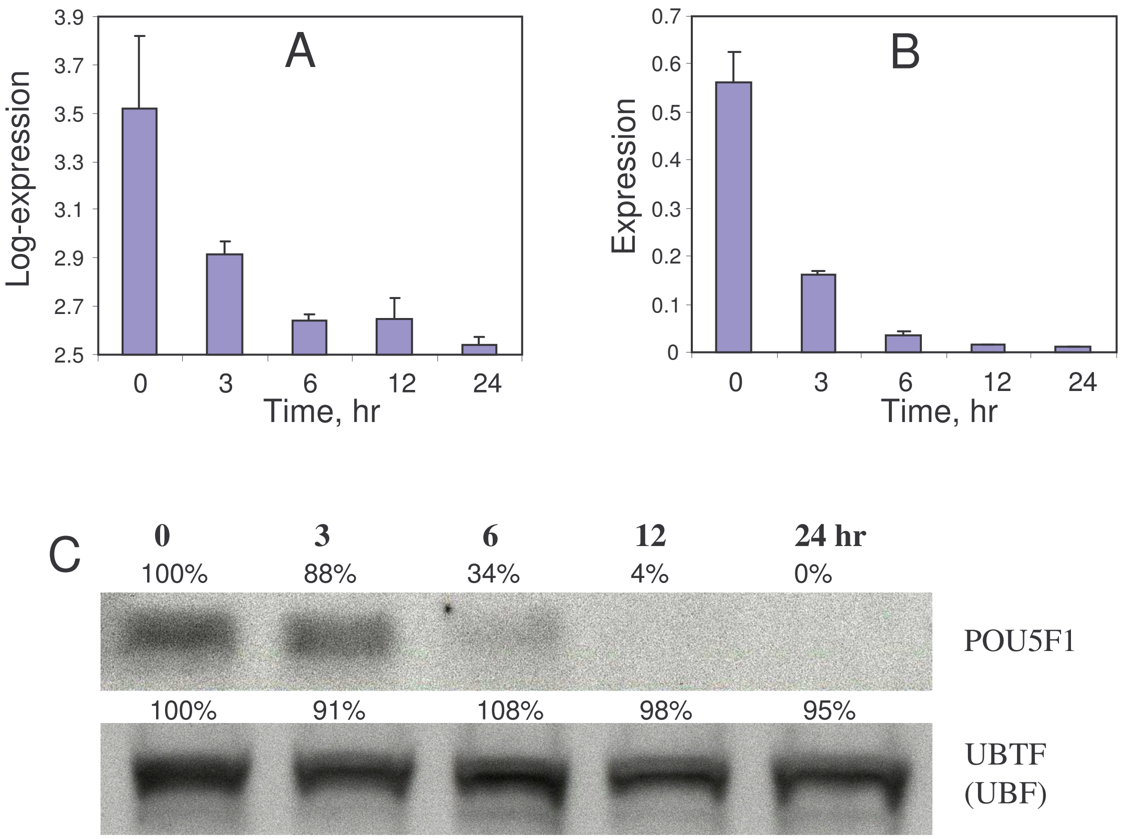

Additional file 3. Suppression of tet-inducible transgene *Pou5f1* in ZHBTc4 ES cells in a time course after adding tetracycline to the media. (A) Log expression change from microarray data (log10), oligo is in ORF. (B) Expression change in real-time PCR data normalized by expression in parental EB5 cell line, primers are in transgene-specific region. (C) Western blot showing decrease in POU5F1 protein amount, UBTF is used as a control.
