# Supplementary material for: Identification of Pou5f1, Sox2, and Nanog downstream target genes with statistical confidence by applying a novel algorithm to time course microarray and genome-wide chromatin immunoprecipitation data
Source: BMC Genomics. 2008 Jun 3;9:269. doi: 10.1186/1471-2164-9-269 (PMC2424064; doi:10.1186/1471-2164-9-269)
Supplement: Additional file 5 — Comparison of genes that responded to Pou5f1 suppression in various studies. (A) Venn diagram of genes that responded to Pou5f1 suppression by > 2 fold in our experiment with tet-inducible ES cell line ZHBTc4 and in published studies of [5,1], which used shRNA as a method of Pou5f1 suppression. (B) Scatter-plot of gene expression change after Pou5f1 suppression in this study and in [1] based on 1225 common genes. [file 1471-2164-9-269-S5.pdf]

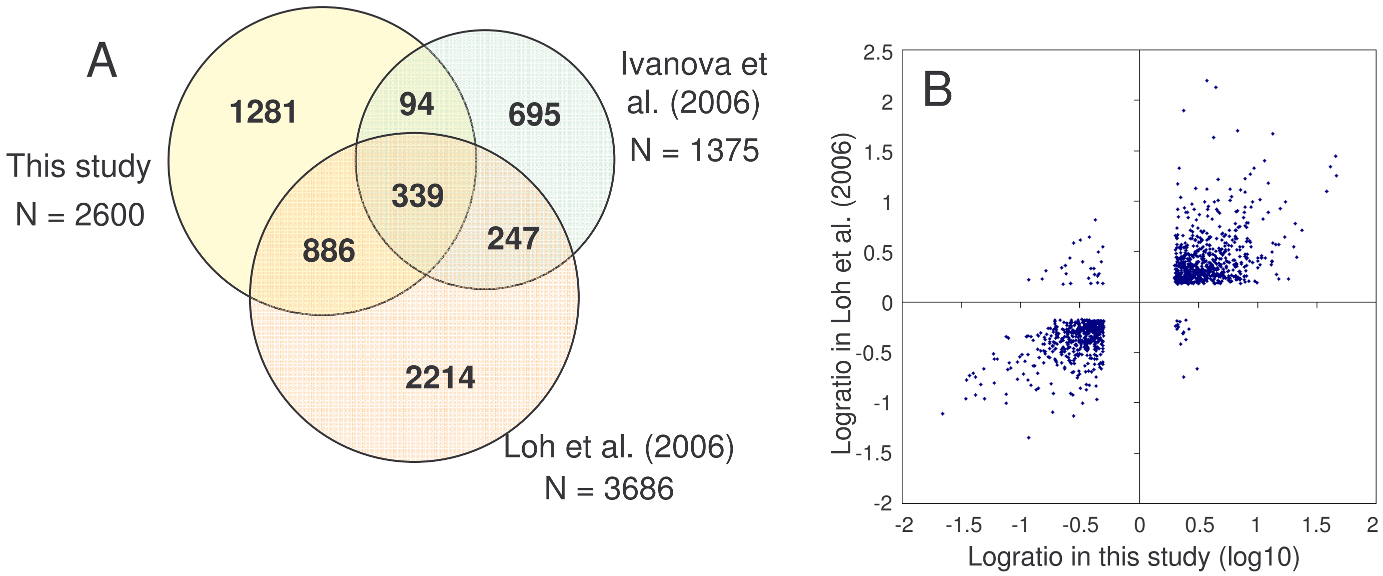

Additional file 5. Comparison of genes that responded to *Pou5f1* suppression in various studies.

(A) Venn diagram of genes that responded to *Pou5f1* suppression in our experiment (>2 fold) with tet-inducible ES cell line ZHBTc4 and in published studies of [1,5], which used shRNA as a method of *Pou5f1* suppression. (B) Scatter-plot of gene expression change after *Pou5f1* suppression in this study and in [1] based on 1225 common genes.
