# Supplementary material for: Identification of Pou5f1, Sox2, and Nanog downstream target genes with statistical confidence by applying a novel algorithm to time course microarray and genome-wide chromatin immunoprecipitation data
Source: BMC Genomics. 2008 Jun 3;9:269. doi: 10.1186/1471-2164-9-269 (PMC2424064; doi:10.1186/1471-2164-9-269)
Supplement: Additional file 10 — Co-localization of POU5F1 and NANOG binding sites in the mouse genome based on ChIP-PET data from [1]. (A) Proportion of ChIP-POU5F1 regions with different number of ditags that were co-localized (withn 1 Kb) with ChIP-NANOG regions (number of ditags indicates binding strength); (B) Density distribution of OCT-SOX composite binding motifs at various distances from the binding site identified with ChIP-PET [1] estimated for groups of genes with ≥ 4 POU5F1 ditags, with ≥ 4 NANOG ditags, and with ≥ 4 NANOG ditags and no POU5F1 ditags. Search for binding motifs was done using components of CisView software [17], which combines pattern-matching and position-weight matrix (PWM) approaches. PWM for OCT-SOX composite binding site was taken from [1]. [file 1471-2164-9-269-S10.pdf]

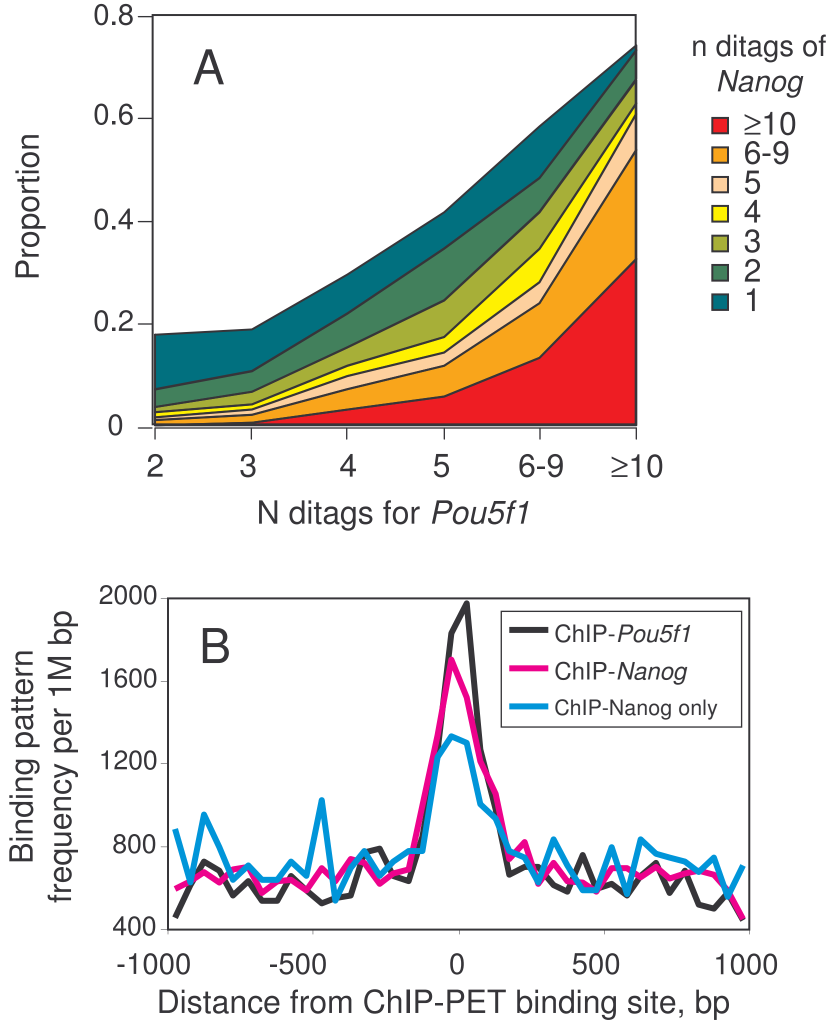

Additional file 10. Co-localization of POU5F1 and NANOG binding sites in the mouse genome based on ChIP-PET data from [1].
(A) Proportion of ChIP-POU5F1 regions with different number of ditags that were co-localized (withn 1 Kb) with ChIP-NANOG regions (number of ditags indicates binding strength); (B) Density distribution of OCT-SOX composite binding motifs at various distances from the binding site identified with ChIP-PET [1] estimated for groups of genes with ≥4 POU5F1 ditags, with ≥4 NANOG ditags, and with ≥4 NANOG ditags and no POU5F1 ditags. Search for binding motifs was done using components of CisView software [17], which combines pattern-matching and position-weight matrix (PWM) approaches. PWM for OCT-SOX composite binding site was taken from [1].
