# Supplementary material for: Identification of Pou5f1, Sox2, and Nanog downstream target genes with statistical confidence by applying a novel algorithm to time course microarray and genome-wide chromatin immunoprecipitation data
Source: BMC Genomics. 2008 Jun 3;9:269. doi: 10.1186/1471-2164-9-269 (PMC2424064; doi:10.1186/1471-2164-9-269)
Supplement: Additional file 15 — Regression of the cumulative proportion of control genes (p) versus the score of potential function (SPF) of their binding regions identified with ChIP-PET [1]. Cumulative proportion of genes was estimated after sorting them by decreasing SPF. Magenta line shows the regression estimated using top 300 genes with highest SPF. (A) SPF parameters estimated by optimization against down-regulated genes; (B) SPF parameters estimated by optimization against up-regulated genes. [file 1471-2164-9-269-S15.pdf]

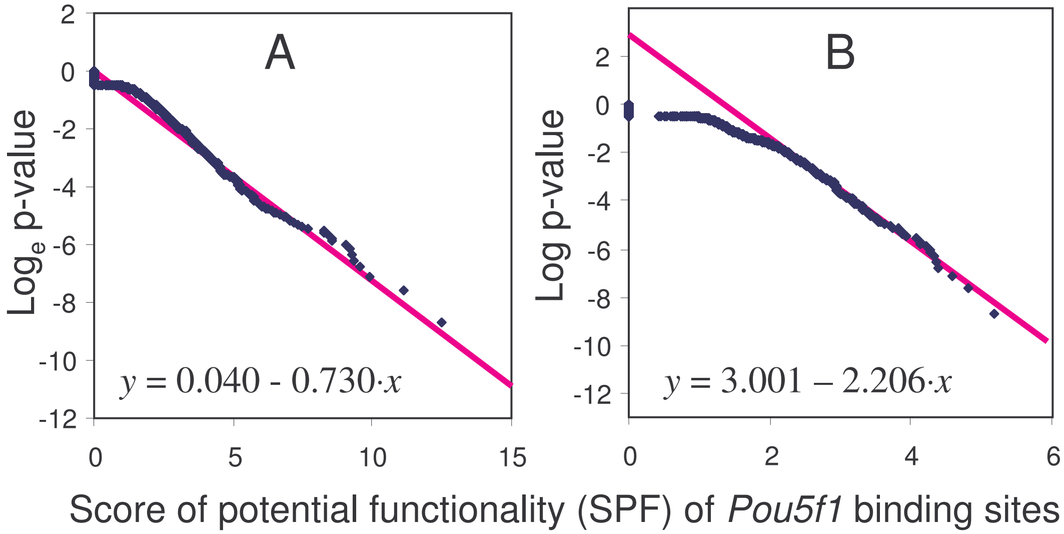

Additional file 15. Regression of the cumulative proportion of control genes (p) versus the score of potential function (SPF) of their binding regions identified with ChIP-PET [1].

Cumulative proportion of genes was estimated after sorting them by decreasing SPF. Magenta line shows the regression estimated using top 300 genes with highest SPF. (A) SPF parameters estimated by optimization against down-regulated genes; (B) SPF parameters estimated by optimization against up-regulated genes.
