# Supplementary material for: Identification of Pou5f1, Sox2, and Nanog downstream target genes with statistical confidence by applying a novel algorithm to time course microarray and genome-wide chromatin immunoprecipitation data
Source: BMC Genomics. 2008 Jun 3;9:269. doi: 10.1186/1471-2164-9-269 (PMC2424064; doi:10.1186/1471-2164-9-269)
Supplement: Additional file 18 — Frequency distribution of a score [SPF·abs(logratio)] among tentative target genes TTGs of POU5F1 identified in this paper and TTGs (non-matching with ours) from [1,2] [file 1471-2164-9-269-S18.pdf]

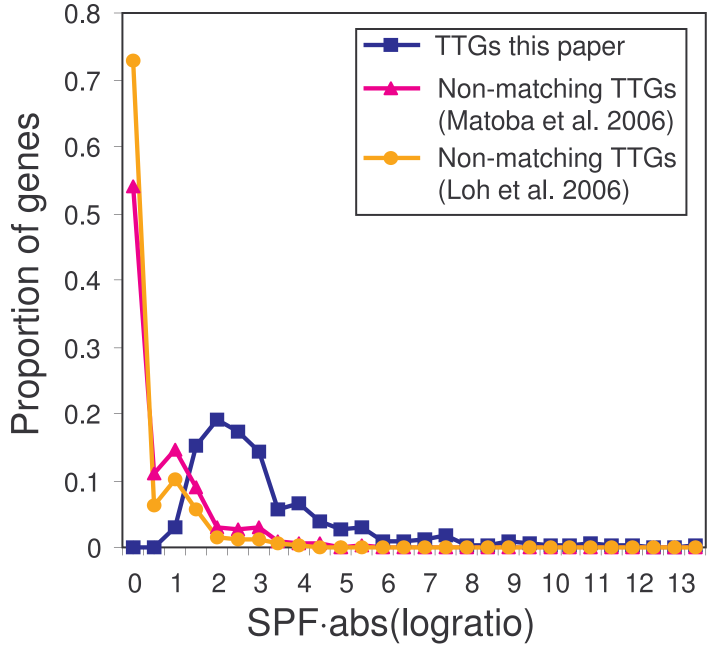

Additional file 18. Frequency distribution of a score [SPF·abs(logratio)] among tentative target genes TTGs of POU5F1 identified in this paper and TTGs (non-matching with ours) from [1,2]
