# Supplementary figures and images for: Identification of Pou5f1, Sox2, and Nanog downstream target genes with statistical confidence by applying a novel algorithm to time course microarray and genome-wide chromatin immunoprecipitation data
Source: BMC Genomics. 2008 Jun 3;9:269. doi: 10.1186/1471-2164-9-269 (PMC2424064; doi:10.1186/1471-2164-9-269)

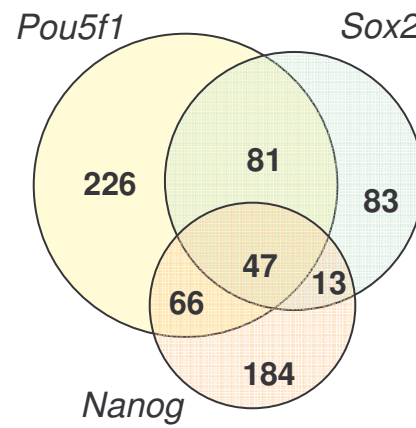

**Additional file 20. Venn diagram of tentative target genes (TTGs) of POU5F1, SOX2, and NANOG**

Supplement: Additional file 20 — Venn diagram of tentative target genes (TTGs) of POU5F1, SOX2, and NANOG [file 1471-2164-9-269-S20.pdf]
