# Supplementary material for: Identification of Pou5f1, Sox2, and Nanog downstream target genes with statistical confidence by applying a novel algorithm to time course microarray and genome-wide chromatin immunoprecipitation data
Source: BMC Genomics. 2008 Jun 3;9:269. doi: 10.1186/1471-2164-9-269 (PMC2424064; doi:10.1186/1471-2164-9-269)
Supplement: Additional file 23 — Expression change of Cdx2 after suppression of Pou5f1 and Sox2 analyzed using quantitative RT-PCR (SE estimated with ANOVA) [file 1471-2164-9-269-S23.pdf]

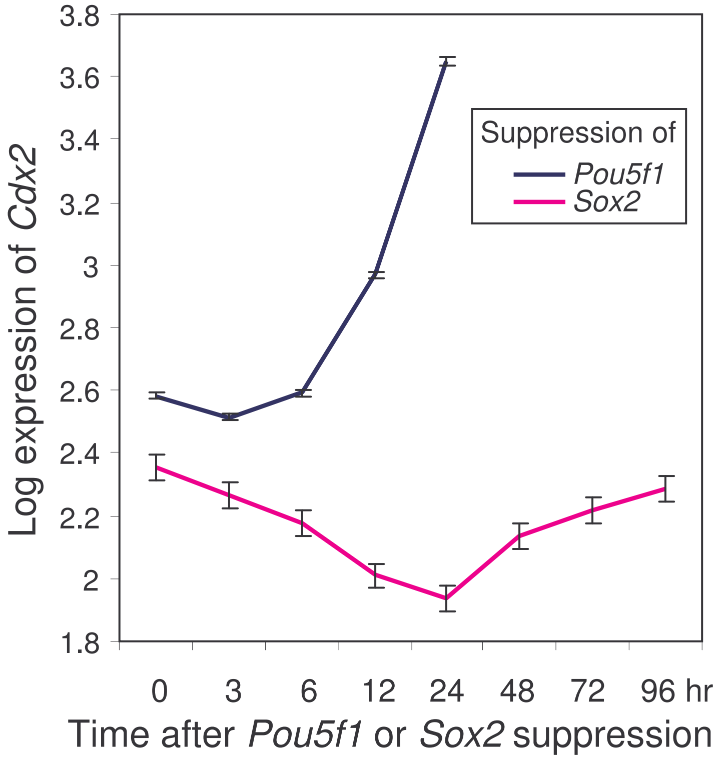

Additional file 23. Expression change of *Cdx2* after suppression of *Pou5f1* and *Sox2* analyzed using quantitative RT-PCR (SE estimated with ANOVA)
